# Promise of combined hydrothermal/chemical and mechanical refining for pretreatment of woody and herbaceous biomass

**DOI:** 10.1186/s13068-016-0505-2

**Published:** 2016-04-30

**Authors:** Sun Min Kim, Bruce S. Dien, Vijay Singh

**Affiliations:** Department of Agricultural and Biological Engineering, University of Illinois at Urbana-Champaign, Urbana, IL 61801 USA; Bioenergy Research Unit, Agricultural Research Service, USDA, National Center for Agricultural Utilization Research, Peoria, IL 61604 USA

**Keywords:** Combined pretreatment, Chemical pretreatment, Hydrothermal pretreatment, Mechanical refining, Lignocellulosic biofuel

## Abstract

Production of advanced biofuels from woody and herbaceous feedstocks is moving into commercialization. Biomass needs to be pretreated to overcome the physicochemical properties of biomass that hinder enzyme accessibility, impeding the conversion of the plant cell walls to fermentable sugars. Pretreatment also remains one of the most costly unit operations in the process and among the most critical because it is the source of chemicals that inhibit enzymes and microorganisms and largely determines enzyme loading and sugar yields. Pretreatments are categorized into hydrothermal (aqueous)/chemical, physical, and biological pretreatments, and the mechanistic details of which are briefly outlined in this review. To leverage the synergistic effects of different pretreatment methods, conducting two or more pretreatments consecutively has gained attention. Especially, combining hydrothermal/chemical pretreatment and mechanical refining, a type of physical pretreatment, has the potential to be applied to an industrial plant. Here, the effects of the combined pretreatment (combined hydrothermal/chemical pretreatment and mechanical refining) on energy consumption, physical structure, sugar yields, and enzyme dosage are summarized.

## Background

Advanced biofuels are advantageous for mitigating greenhouse gas emissions associated with transportation and for promoting rural development [1]. Encouraged by governmental policies, commercial facilities have begun producing second-generation ethanol in several countries. The first modern commercial facility is located in Italy and began operation in January 2013. In the United States, biochemical-based facilities include POET-DSM (Emmetsburg IA) opened in September 2014, Quad County Corn Processors (Galva, IA) opened in September 2015, and E. I. du Pont de Nemours and Company (Nevada, IA) opened in October 2015.

**Fig. 1:**
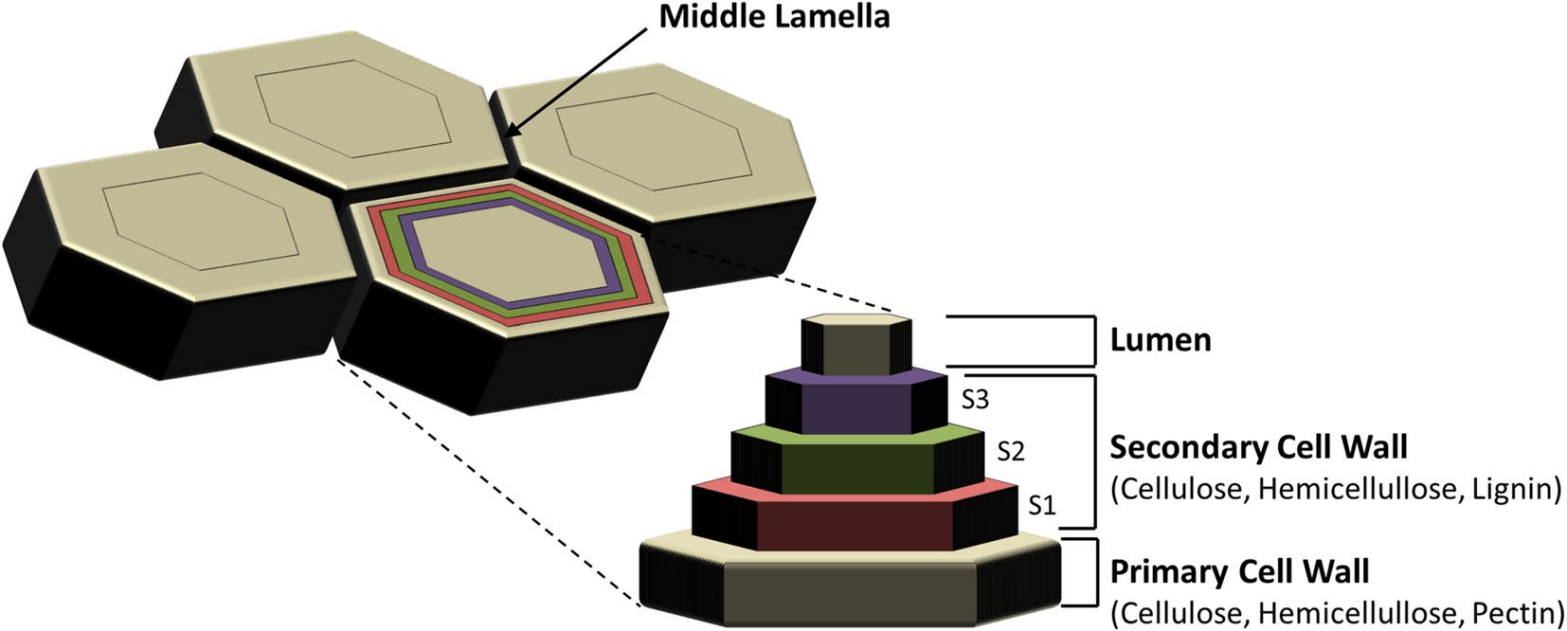
Plant structure consisting of primary and secondary cell walls, lumen, and middle lamella

Within the United States, it is estimated that upward of one billion tons of biomass could be produced each year [2]: enough to substitute for one-third of domestic petroleum usage. Major sources of lignocellulosic biomass include agriculture residues; pulp, paper, and forestry industrial waste; and food processing and municipal solid wastes. Additional biomass is potentially available through the production of dedicated bioenergy crops including tree plantations and perennial grasses. The structure of the plant cell wall is depicted in Fig. 1. Cellulose and hemicellulose are in the primary and secondary cell walls. Databases have been made available that list the chemical composition for various sources of biomass [the Biomass Feedstock Composition and Property Database offered by the U.S. Department of Energy (DOE) (http://www.afdc.energy.gov/biomass/progs/search1.cgi) and Energy Research Centre of the Netherlands’ Phyllis2 (https://www.ecn.nl/phyllis2/Browse/Standard/ECN-Phyllis#)]. According to the U.S. DOE database (Table 1), agriculture residues contain 31–43 % cellulose, 12–25 % hemicellulose, and 17–24 % total lignin. Hardwoods have 36–49 % cellulose, 14–23 % hemicellulose, and 17–29 % total lignin. Herbaceous dedicated energy crops have 30–38 % cellulose, 16–26 % hemicellulose, and 16–25 % total lignin.

**Table 1:** Biomass feedstock composition

Biomass feedstock	Cellulose (%)	Hemicellulose (%)	Lignin (%)
Agriculture residues	31–43	12–25	17–24
Hardwoods	36–49	14–23	17–29
Herbaceous dedicated energy crops	30–38	16–26	16–25

(US DOE database: http://www.afdc.energy.gov/biomass/progs/search1.cgi)

**Fig. 2:**
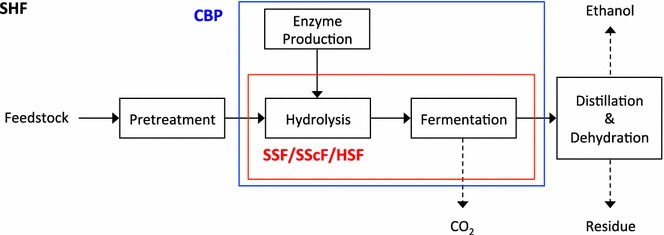
Cellulosic ethanol process. *SHF* separate hydrolysis and fermentation. *SSF* simultaneous saccharification and fermentation. *SScF* simultaneous saccharification and co-fermentation. *HSF* hybrid saccharification and fermentation. *CBP* consolidated bioprocessing. Adapted from [71]

**Fig. 3:**
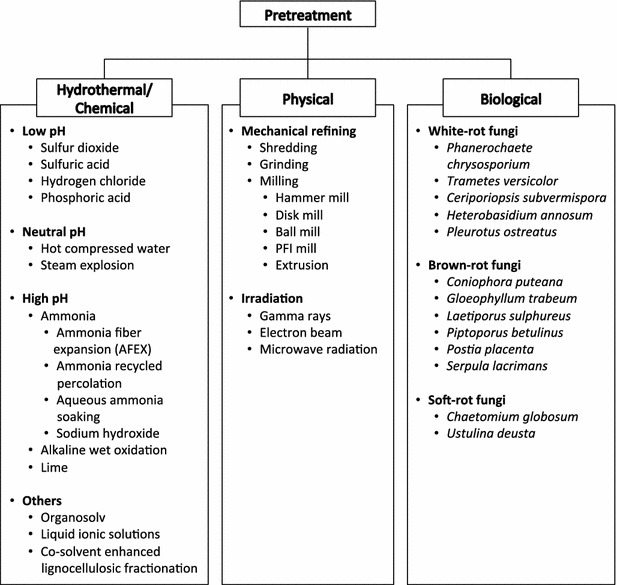
Types of pretreatment

The lignocellulosic ethanol production process is depicted in Fig. 2. First, feedstock is transported into a plant and ground to a mean size of 0.16–0.23 in (0.41–0.58 cm) [3]. The next step in the lignocellulosic ethanol production process is pretreatment. The recalcitrance of biomass, which is caused by epidermal tissue and chemicals (cuticle, wax, and bark), composition (lignin, hemicellulose, and pectin), the physical structure of the cell wall (heterogeneity and complexity), cellulose structure (crystallinity), and pretreatment-induced effects (cellulose re-annealing and melted lignin), prevents enzyme accessibility to cellulose [4]. To reduce biomass recalcitrance and increase enzyme accessibility to cellulose, pretreatment that disrupts the biomass cell walls is necessary. Pretreatment can be done by hydrothermal/chemical, physical, and biological methods (Fig. 3).

Hydrolysis and fermentation follow pretreatment, and can be done by separate hydrolysis and fermentation (SHF), simultaneous saccharification and fermentation (SSF), simultaneous saccharification and co-fermentation (SScF), hybrid saccharification and fermentation (HSF), or consolidated bioprocessing (CBP). For SHF, hydrolysis and fermentation can each be performed at optimal conditions, and yeast either can be recycled or possibly collected and marketed for feed. However, there are end-product inhibitions and sugar losses during lignin separation before fermentation, both of which result in decreased ethanol yields [5, 6]. To reduce capital investment costs, hydrolysis and fermentation operations can be combined in a single reactor for the SSF process. Ethanol yields are higher in SSF compared to SHF, but more enzymes are required and yeast cannot be reused [5, 6]. When engineered yeasts that can ferment C5 and C6 sugars are used in the SSF, the process is termed as SScF. Adding surfactant, such as Tween 80, increases ethanol yield, reducing enzyme loading and increasing enzyme activity by preventing unproductive binding of the cellulases to lignin in SSF and SHF [7, 8]. Even though many studies have shown that SSF produced higher ethanol yields than SHF [9, 10], there are few reports showing higher ethanol yields from SHF than SSF [11–13]. To leverage SHF and SSF, HSF has been developed. In HSF, samples are incubated with cellulase at its optimal conditions, and then SSF is performed. The basis of CBP is to use microorganisms that produce the needed hydrolysis enzymes. While it offers great promise for the future, this scheme is still in the research stage [14, 15].

Following fermentation, ethanol is recovered by distillation and subsequently dehydrated using molecular sieves to break the 95 % azeotrope. The residual solids are recovered from the bottom of the distillation column and then moved to a combustor to generate electricity [3].

Each step in the advanced ethanol process plays an important role, but pretreatment is a critical step in the process [16, 17]. The choice of a pretreatment method affects downstream processes including conditioning of pretreated samples, enzyme formulation and dosage, microorganism selection, recovery of co-products, and wastewater treatment [16, 17]. It is important to integrate pretreatment design and operation with the whole process.

In this regard, two-stage pretreatments that combine hydrothermal and/or chemical followed by mechanical refining are of particular promise for both woody and herbaceous biomass. As will be discussed, a two-stage pretreatment can often afford higher product yields and offers significant advantages for the other unit operations. This review briefly outlines the mechanistic details of available pretreatment methods. Also, the effects of the combined pretreatment (hydrothermal/chemical pretreatment with mechanical refining) are summarized, including effects on sugar yields, enzyme dosage, energy consumption, size reduction, and crystallinity. Lastly, potential commercial-scale application of the combined pretreatment will be discussed.

## One-step pretreatment

An in-depth review of biological and chemical pretreatments will not be discussed here because these have been the subject of numerous recent reviews [18–20]. Readers with particular interest in such chemical pretreatment as organosolv pretreatment [21, 22], liquid ionic solution pretreatment [23, 24], and co-solvent enhanced lignocellulosic fractionation (CELF) [25] are directed to the respective references cited. A full list of pretreatments classified by mechanism is shown in Fig. 3. Only those pretreatments relevant to combined systems will be discussed here, which include dilute acid, hydrothermal and alkaline pretreatments, and mechanical refining.

### Chemical pretreatment

Various chemicals with a wide range of pH are used to pretreat biomass (Fig. 3). Depending on pH, different pretreatment kinetic models have been proposed. Low pH pretreatments, including sulfur dioxide, sulfuric acid, and hydrochloric acid hydrolyze most of the hemicellulose [26]. One proposed hemicellulose kinetic model states that hemicellulose is solubilized to xylose oligomers from fast and slow reactions (biphasic hemicellulose hydrolysis) [27]. Then, xylose monomers are produced, which are further reduced to furfural. Cellulose is hydrolyzed to insoluble oligomers that cannot be hydrolyzed further and to soluble oligomers that are hydrolyzed to monomers. Glucose monomers are further reduced to hydroxymethylfurfural (HMF) [28]. Low pH pretreatments have been extensively studied, and dilute acid-pretreated samples have shown high enzyme digestibility. However, the primary disadvantages of acid pretreatment are the generation of inhibitors to enzymes and yeasts, and the requirement of corrosion-resistant reactors [29].

Pretreatments at high pH include ammonia, sodium hydroxide, and alkaline wet oxidation. Ammonia is used in many methods, such as ammonia recycled percolation (ARP), aqueous ammonia soaking (AAS), and ammonia fiber explosion (AFEX). Alkaline pretreatment mainly entails delignification, which has three stages: initial, bulk, and terminal (residual) [30, 31]. The initial stage occurs at low activation energy (61 kJ/mol) and temperature (<170 °C), where α-aryl ether and β-aryl ether bonds in phenolic units are cleaved rapidly. The bulk stage occurs at high activation energy (150 kJ/mol) and temperature (170 °C), where non-phenolic β-aryl ether linkages are cleaved. At the last stage, the terminal or residual delignification, cleavage of C–C linkages, and degradation of carbohydrates take place. A drawback of alkaline pretreatment is that it generates irrecoverable salts, which penetrate into biomass [32]. Also, it is not effective to use in high-lignin-content biomass such as softwoods, even though it is effective on herbaceous biomass, hardwood, and agriculture residues [32].

### Hydrothermal (aqueous) pretreatment

Pretreatment at neutral pH is an acid catalyzed process. At high temperature and pressure, saturated liquid water increases the concentration of protons in solution, becoming weakly acidic. H^+^ and OH^−^ concentrations in water at 250 °C are 23.3 times higher than those at 25 °C. In addition, hemicellulose is hydrolyzed in acidic conditions and releases acetyl and uronic groups. These acids, especially acetic acid, hydrolyze links between hemicelluloses and lignin, which is the reason that aqueous pretreatment is named autohydrolysis [33]. Examples of autohydrolysis are liquid hot water pretreatment and steam explosion. Liquid hot water pretreatment is also termed as hot water pretreatment, hot compressed water pretreatment, and hydrothermal pretreatment. Since the pretreatment does not use any other chemicals, it is an environmentally friendly method with low operation and capital costs compared to chemical pretreatment [34]. However, hot water pretreatment requires 20–50 °C higher temperatures and 5–15 min longer residence times compared to dilute acid pretreatment to gain the same cellulose conversion yields. Hot water also puts greater demand on the cellulase/hemicellulase enzyme system because, unlike dilute acid, it does not end-saccharify the hemicellulose carbohydrates.

### Mechanical refining

Mechanical refining includes shredding, grinding, and milling [35], which reduce particle size and increase the available specific surface area for hydrolysis. The three main roles of mechanical refining are cutting (shortening), shearing (external fibrillation), and compression (internal fibrillation) [36]. Plant cell walls consist of primary and secondary layers (Fig. 1). By shearing, outer walls of fiber are pulled out and primary walls are removed. Compression breaks intrafiber hydrogen bonds and reforms the bonds with water molecules, which causes internal fibrillation [36].

**Fig. 4:**
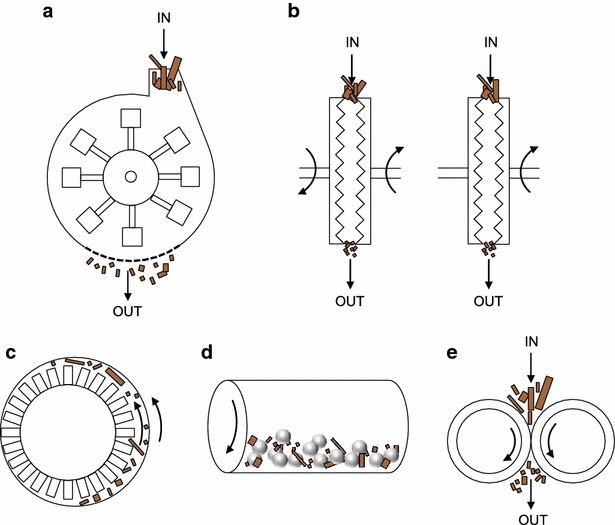
Types of mill for biomass pretreatment. **a** hammer mill; **b** disk mill; **c** PFI mill (laboratory testing only); **d** ball mill; **e** roller mill

**Fig. 5:**
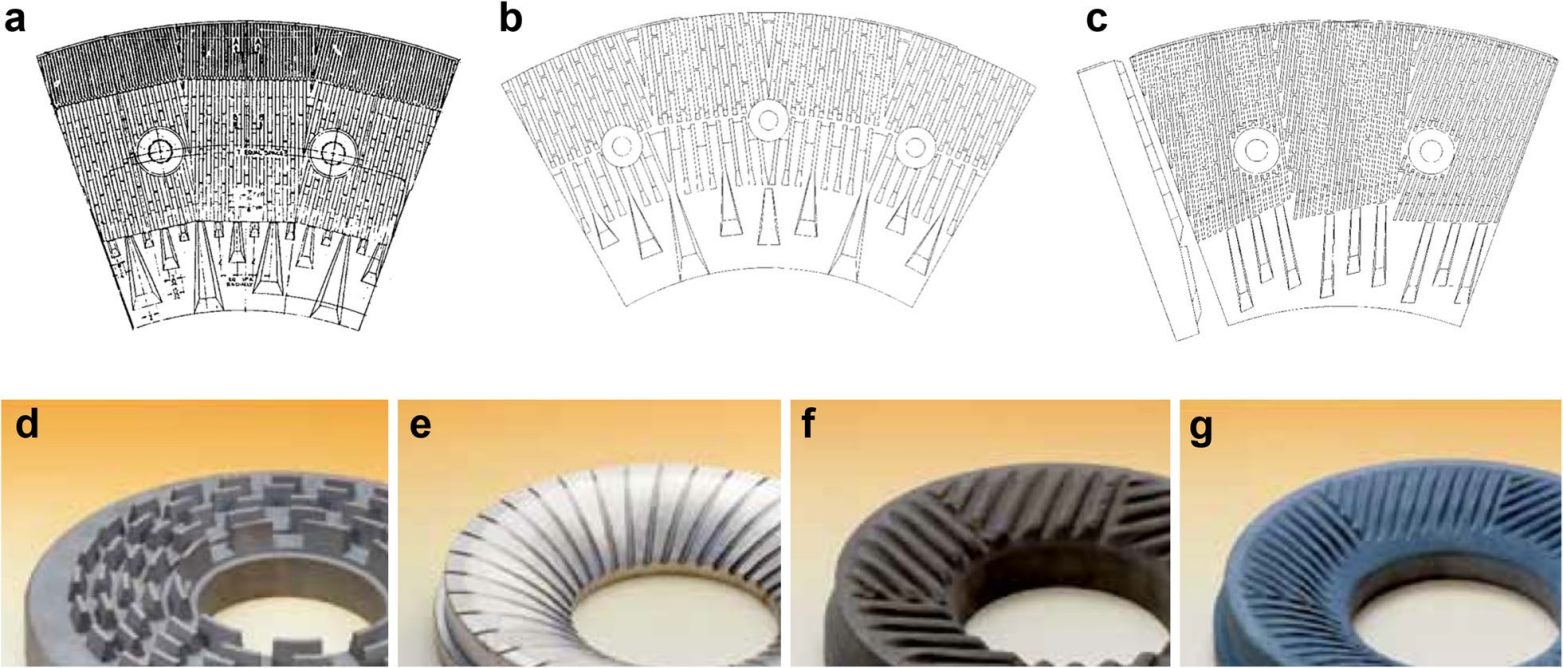
Disk mill plate designs: **a** Fine bidirectional pattern (Durametal D14-002); **b** coarse bidirectional pattern (Durametal 36,602) [74]; **c** directional pattern (Durametal 36,604); **d**–**g** Granomat disk mills (brochure from Fuchs Maschinen AG)

The choice of mill for mechanical pretreatment can be determined based on biomass moisture content. Knife mills and hammer mills are suitable for dry samples but do little to disrupt cell walls and are generally not used for pretreatment purposes, but are important for reducing particle sizes to increase biomass flowability (Fig. 4). Ball mills, extruders, and disk (disk) mills are the major scalable methods used for pretreatment. These unit operations are scalable and adapted for dry and wet samples (Fig. 4). Ball mills grind using shear and compressive forces. Ball milling reduces cellulose crystallinity as well as particle size [37, 38]. Since the ball milling can be done with high slurry concentration, it reduces reactor volume and capital costs. However, long milling times and high processing costs, including power usage, can make ball milling impractical on an industrial scale [39]. Extruders provide shear force, heating, and mixing, which can achieve thermomechanical and chemical pretreatments at the same time. Single-screw and twin-screw extruders have been widely studied for biomass pretreatment [40, 41]. However, screw extrusion requires a high energy input and capital investment, which might prevent practical industrial-scale application [42]. Disk mills consist of two grooved disks: either counter-rotating disks, or one stationary and one rotating disk. Disk milling is a continuous process and mainly utilizes shear force to induce biomass defiberization [43, 44]. Disk milling is scalable but has high energy consumption [45]. Examples of disk mills that are used in lignocellulosic ethanol processing are summarized in Table 2, and various disk mill plate designs are depicted in Fig. 5. Disk mill plates are designed with both bars and grooves. The leading edges of the bars impact fibers, while the grooves determine the capacity of the mill [46]. To grind chemically pretreated biomass, mills require special materials that resist corrosion. Table 3 summarizes potential mill materials that have low corrosive rates when in contact with sulfuric acid [47].

**Table 2:** Disk mills used in cellulosic ethanol processing

Model	Power (kW)	Diameter (inch)	Throughput (kg/hr)	Company	Reference
Sprout Waldron disk mill (Model: 12-1CP)	44.8	12	1−5 oven dried kg/run	Koppers Company, Inc. (Muncy, PA)	[53]
Beloit double-disk (Model: 4342HS)	112	42	10,000−20,000	Beloit (Dalton, MA)	[53]
Sprout-Bauer twin flow refiner		42	10,000−20,000	Andritz Sprout-Bauer (Muncy, PA)	[53]
Sprout 401 double disk refiner	224	36	NR	Andritz Sprout-Bauer (Muncy, PA)	[42]
Lab disk mill: 12″	37.3	12	NR	Andritz Sprout-Bauer (Muncy, PA)	[49, 52]
KRK continuous high-consistency refiner (No. 2500-II)	30	12	NR	Kumagai Riki Kogyo Co, Ltd. (Tokyo, Japan)	[65]
Supermasscolloider (Model: MKZA10)	15	9.84	80–1200	Masuko Sangyo Co, Ltd. (Saitama, Japan)	[56, 60, 72]
Supermasscolloider (Model: MKCA6-2)	1.50	5.9	35−120	Masuko Sangyo Co, Ltd. (Saitama, Japan)	[54, 63, 73]

*NR* means not reported

**Table 3:** Potential materials of construction for mills to grind dilute acid-pretreated samples. Corrosion rate tests were performed at the sulfuric acid boiling temperature [47]

Material	Condition, other factors and comments	Concentration (%)	Duration (h)	Corrosive rate (mm/year)
Irons and steels				
Altemp A-286	Solution treated	10	NR^a^	0.75
Stainless steels				
AL 29-4-2	Dilute^b^	10	NR	0.46
Altemp 625	Dilute^b^	10	NR	0.64
E-Brite	Dilute, nonactivated	5	48	0.356
Type 316 stainless steel	NR	0.25	24	0.0686
Titanium				
Ti-3-8-6-4-4	Plus 1 g/L FeCl_3_	10	NR	0.15
Ti-3A1-2.5 V	ASTM grade 9	0.5	NR	0.35
Titanium	Grade 7 plus 16 g/L Fe_2_(CO_4_)_3_	10	NR	0.178
Titanium	Grade 12. naturally aerated	1	NR	0.91
Tantalum				
Tantalum	NR	10	NR	<0.02
Alloys				
Alloy C-22	NR	20	NR	0.838
Alloy C-4	NR	10	NR	0.787
Alloys				
Ferralium	NR	5	NR	0.30
Hastelloy B	SO_2_ purge	10	NR	0.05–0.25
Hastelloy B-2	SO_2_ purge	10	NR	0.05–0.25
Hastelloy C	Lab test	10	120	>0.25–0.51
Hastelloy G	Lab test	10	120	>0.25–0.51
Hastelloy G-3	Lab test	10	120	>0.25–0.51
Hastelloy G-30	Plus 42 g/L Fe_2_(SO_4_)_3_	50	NR	0.171
Hastelloy G-30	Plus 10 % nitric acid	50	NR	0.406
Inconel 617	Average of two tests	5	NR	0.61
Others				
Columbium^c^		10	NR	<0.12
Niobium	NR	10	NR	0.125

^a^NR means not reported

^b^Activated before tests

^c^Susceptible to embrittlement

Papirindustriens Forskningsinstitutt (PFI) mills are a specialty type of shear mill developed for laboratory-scaled paper pulp testing that have proven valuable for mechanical pretreatment studies. A PFI mill consists of bars and a smooth bedplate [48]. The bars are pushed to one side of the bedplate, which provides compression and shear forces to fibers. Compression is the main force of PFI milling that causes internal fibrillation. While PFI mills are suitable for laboratory studies, they are not scalable for continuous units [49].

### Combined pretreatment: hydrothermal/chemical pretreatment with mechanical refining

While single-stage pretreatments dominate the biomass conversion literature, this is not the case in pulping where processes combing chemical/hydrothermal processing with mechanical refining dominate [50, 51]. The trend toward two-stage pretreatments is a recent phenomenon for biomass conversion as shown by the studies listed in Table 4. For pretreatment of woody biomass, the successful strategies [>85 % conversion to sugar(s)] have involved dilute acid (with or without sulfite addition), ozonolysis, alkaline, and hydrothermal using either hot water or steam all followed by disk milling. Ball milling, preceded by alkaline pretreatment, has only been described for oil palm mesocarp. In the case of herbaceous biomass, there are fewer studies, and most have pretreated corn stover using alkali deacetylation followed by dilute acid, and mechanical refining using either a roller (Szego) or disk-type mills. Rice straw has also been successfully converted solely using hot water followed by disk milling. Sugarcane bagasse was successfully converted using hot water followed by PFI refining; PFI mills are marketed for laboratory-scaled testing of pulp quality. Disapprovingly there have been no studies using perennial grasses. The emphasis on wood is not unexpected because (as will be described) the energy requirement for particle reduction is much higher than for herbaceous biomass, and this can be dramatically reduced by prior delignification. Two-stage processes using wood feedstocks can also be readily scaled-up because of experience in pulping.

**Table 4:** Comparison of hydrothermal/chemical pretreatment followed by mechanical refining and hydrothermal/chemical pretreatment alone or mechanical refining alone

Sample	Pretreatment^a^	Milling energy (kWh/ton)^b, c^	Sugar yield (%)^d^	Reference
Hardwood chips	Sodium carbonate	NA^e^	42.11 (total sugar)	[53]
	Sodium carbonate + PFI milling	360–1800	46.90–53.12 (total sugar)	
	Sodium carbonate + disk milling (12 inch diameter)	698	69.51 (total sugar)	
	Sodium carbonate + disk milling (42 inch diameter)	67–147	62.48–66.51 (total sugar)	
Japanese cedar	Ozonolysis	NA	28–68 (glucose)	[54]
	Disk milling	4167–26,389	29–44 (xylose)	
	Ozonolysis + disk milling	8333–22,222	38–75 (glucose)	
			26–45 (xylose)	
			71–94 (glucose)	
			44–59 (xylose)	
Lodgepole pine trees	Disk milling	615.9	11.3 (glucose)	[52]
	Hot water (initial pH 5.0) + disk milling	537.0	33.1 (glucose)	
	Acid (initial pH 1.1) + disk milling	335.6	39.6 (glucose)	
	SPORL (initial pH 4.2) + disk milling	499.3	84.1 (glucose)	
	SPORL (initial pH 1.9) + disk milling	134.5	92.2 (glucose)	
Eucalypt chips	Disk milling	990	72.94 (total sugar)	[65]
	Sodium hydroxide impregnation + disk milling	630	80.77 (total sugar)	
	Magnesium hydroxide impregnation + disk milling	430	91.53 (total sugar)	
Hinoki cypress eucalyptus chips	Disk milling	853	50 (glucose)	[73]
	Steam treatment + disk milling	744–1489	96.8 (glucose)	
Eucalyptus chips	Disk milling	408	45 (glucose)	
	Steam treatment + disk milling	192–458	98.4 (glucose)	
Eucalyptus chips	Hot water	NA	50 (glucose)	[55]
	Hot water + disk milling	167	101.7 (glucose)	
Eucalyptus chips	Hot water	NA	3.1–65.2 (total sugar)	[37]
	Hot water + ball milling	1436	45.6–66.7 (total sugar)	
Rice straw	Hot water	NA	97.5 (glucose)	[62]
	Hot water + mechanical refining	250–583	97.3–99.5 (glucose)	
Oil palm mesocarp fiber	Disk milling	5250	30.2 (glucose)	[56]
	Superheated steam + disk milling	1417–3028	30.6 (xylose)	
	Hot water + disk milling	4083–4972	26.0–47.8 (glucose)	
			24.1–42.1 (xylose)	
			46.3–91.1 (glucose)	
			10.1–54.3 (xylose)	
Corn stover	Alkali deacetylation + disk milling (36 inch diameter)	128–468	85.9–91.7 (glucose)	[42]
			81.1–86.2 (xylose)	
Sugarcane bagasse	Alkaline + disk milling	11,111	77 (glucose)	[63]
			67 (xylose)	
Sugarcane bagasse	Hot water	NA	72.1–78.7 (total sugar)	[75]
	Hot water + PFI refining		82.1–87.2 (total sugar)	
Wheat straw	Hot water	NA	28.1–72.4 (total sugar)	[59]
	Hot water + PFI refining		28.3–75.5 (total sugar)	
Oil palm mesocarp fiber	Ball milling	NR^f^	7.3–10.3 (glucose)	[38]
	Alkaline		12.2–14.9 (xylose)	
	Alkaline + ball milling		39.6–63.9 (glucose)	
			21.1–46.5 (xylose)	
			97.3 (glucose)	
			63.2 (xylose)	
Corn stover	Acid impregnation + dilute acid	NA	69–73 (glucose)	[58]
	Alkali deacetylation + acid impregnation + dilute acid		55–58 (xylose)	
	Acid impregnation + dilute acid + PFI refining		80–83 (glucose)	
	Alkali deacetylation + acid impregnation + dilute acid + PFI refining		76–80 (xylose)	
			85 (glucose)	
			75 (xylose)	
			90 (glucose)	
			92 (xylose)	
Corn stover	Alkali deacetylation + acid impregnation + steam explosion + PFI refining	NR	79–83 (glucose)	[49]
	Alkali deacetylation + acid impregnation + steam explosion + extruder		50–55 (xylose)	
	Alkali deacetylation + acid impregnation + steam explosion + food processor/blending		82–83 (glucose)	
	Alkali deacetylation + acid impregnation + steam explosion + disk milling (12 inch)		56–58 (xylose)	
	Alkali deacetylation + acid impregnation + dilute acid (pilot-scale)		71–75 (glucose)	
	Alkali deacetylation + acid impregnation + dilute acid pretreatment (pilot-scale) + Szego milling		49–51 (xylose)	
			75–78 (glucose)	
			52–54 (xylose)	
			82 (glucose)	
			80 (xylose)	
			90–95 (glucose)	
			85–90 (xylose)	
Eucalyptus chips	Hot water	NA	73.19 (glucose)	[72]
	Hot water + disk milling		90.45 (xylose)	
			91.62 (glucose)	
			88.12 (xylose)	
Rice straw	Disk milling	NR	86 (glucose)	[60]
	Hot water + disk milling		40 (xylose)	
			110 (glucose)	
			84 (xylose)	

^a^Hot compressed water, hydrothermal and autohydrolysis are named as hot water

^b^When energy consumption was presented as kJ/ton, it was converted into kWh/ton

^c^Energy consumption is only from mechanical refining

^d^If the exact sugar yields were not indicated in the reports, sugar yields were estimated or calculated as the ratio of the amount of monosaccharides produced during hydrolysis to the corresponding carbohydrate concentrations in the original samples

^e^NA means not applicable

^f^NR means not reported

### Effects on sugar yields and enzyme dosage

The synergistic effects of combined pretreatment on biomass structure improve sugar yields. Hydrothermal/chemical pretreatment followed by milling improved sugar yields from 1.16–9.45-fold and 1.04–2.03-fold compared to milling alone and hydrothermal/chemical pretreatment alone, respectively (Table 4). Milling after hydrothermal/chemical pretreatment has a huge impact on increasing sugar yields compared to milling alone because of differing effects on the cell wall structure. For example, oil palm mesocarp fiber has a particularly rigid surface, so milling alone was not enough to break the strong cellulose–hemicellulose–lignin network and overcome recalcitrance [38]. Moreover, milling alone was not as effective as hydrothermal/chemical pretreatment alone to increase enzymatic hydrolysis efficiency. Ball-milled oil palm mesocarp fiber showed 10.3 % glucose yield and 14.9 % xylose yield, while alkaline-pretreated sample showed 63.9 % glucose yield and 46.5 % xylose yield. Glucose and xylose yields were improved up to 97.3 and 63.2 %, respectively, by alkaline pretreatment followed by ball milling [38]. In addition, disk milling alone on lodgepole pine trees did not achieve high glucose yield (11.3 %), which was improved to 92.2 % after combined pretreatment (sulfite pretreatment to overcome recalcitrance of lignocellulose (SPORL) with disk milling) [52].

Many types of refining mills have been used to improve sugar yields, including disk, ball, PFI, and roller (Szego) mills as well as extruders. However, it is hard to choose the best type of mill because biomass structure, types of hydrothermal/chemical pretreatment, and milling conditions affect overall sugar yields. For example, in the case of sodium carbonate-pretreated hardwood chips, higher overall sugar yields were achieved after disk milling (69.5 %) than PFI milling (53.1 %) [53]. However, for corn stover that underwent alkali deacetylation, acid impregnation, and steam explosion, PFI milling attained higher glucose yield (83 %) and xylose yield (55 %) than disk milling (78 % glucose yield and 54 % xylose yield) [49].

Sugar yields increase and plateau as milling time increases [54–56]. This is because of internal and external fibrillation. For example, beating disrupts the fiber’s amorphous area, and opens pores [57]. However, in the case of PFI milling, excessive beating eventually decreases enzymatic accessibility by collapsing the micro-pore structure [49, 53, 57]. In one example, when PFI mill revolutions were increased from 8000 to 10,000, enzyme digestibility decreased from ~77 to ~70 % [49].

Hydrothermal/chemical pretreatment followed by mechanical refining achieves high sugar yields with low enzyme dosages [58, 59]. Disk-milled samples showed 71.5 % glucose yield and 49.6 % xylose yield at a cellulase loading of 20 FPU/g rice straw [60]. However, higher glucose yield (86.7 %) and xylose yield (74.4 %) were achieved with lower enzyme dosage (5 FPU/g rice straw) when samples were hot water pretreated and disk milled. Similarly, sugar yields of hot water-pretreated and ball-milled sample at a cellulase loading of 4 FPU/g substrate are comparable with sugar yields of hot compressed water-pretreated sample or ball-milled sample at a cellulase loading of 40 FPU/g substrate [37].

The paper industry has equipment that combines thermal heating and mechanical refining. It can be supposed that refining biomass at temperatures above the melt temperature of lignin might have consequences not observed by refining after the melted lignin has set. The lack of data on what happens when these two elements are combined for herbaceous biomass represents a significant research gap.

### Energy consumption

Mechanical refining is an energy-intensive process that relies on electrical power. Energy balances have been well studied for wood pretreated with the SPORL process, which require greater amounts of energy compared to grass-based crops. For practical application in a lignocellulosic ethanol plant, this energy requirement must be reduced. Since adding chemical or hydrothermal pretreatment after mechanical refining introduces an extra energy requirement, combining pretreatment steps in this order is not economically feasible. For example, for wood chips, about 10–40 % of the ethanol thermal energy from wood (2000 kWh/ton wood) would be consumed during size reduction by mechanical refining, when it precedes hydrothermal/chemical pretreatment. However, to achieve a practical net energy output from wood biomass ethanol, energy consumption for mechanical refining preferably should be in the range of 27.78–111.11 kWh/ton [61]. To reduce mechanical refining energy, hydrothermal/chemical pretreatment before mechanical refining has been suggested [52, 55, 56]. Hydrothermal/chemical pretreatments remove hemicellulose, lignin or both, and produce nanoscopic pores between cellulose microfibril bundles, which weaken the network structure of the polymer matrix [52, 55, 56]. This allows the energy requirement for mechanical refining after hydrothermal/chemical pretreatment to be reduced by up to 80 % compared to mechanical refining alone for wood biomass (Table 4).

There are only a few reports in which milling energy consumption of chemically or hydrothermally pretreated agriculture residues was measured [42, 62, 63]. Milling energy consumption of alkali deacetylated corn stover was 128–468 kWh/ton [42], which was similar to hydrothermally pretreated rice straw (250–585 kWh/ton) [62]. Much higher milling energy was required for alkaline-pretreated sugarcane bagasse (11,111 kWh/ton) [63]. However, unpretreated sample milling energy consumptions were not reported, so the actual benefit of combined pretreatment on reducing milling energy remains unknown for pretreated agriculture residues.

Hydrothermal/chemical pretreatment plays a critical role in mechanical refining energy consumption (Table 4) because different types of chemical pretreatments have unique mechanisms to destruct the cell wall. Four different pretreatment methods (hot water, acid, SPORL with initial pH 4.2, and SPORL with initial pH 1.9) were applied to lodgepole pine wood chips, and disk milling followed [52]. Compared to disk milling alone, hot water pretreatment with disk milling barely reduced energy consumption and only slightly increased substrate enzymatic digestibility. However, SPORL with initial pH 1.9 followed by disk milling saved 78 % of the milling energy and achieved 92.2 % substrate enzymatic digestibility. This is because the SPORL process not only removes hemicellulose, but also sulfonates lignin [64]. Lignin becomes hydrophilic after sulfonation, which promotes swelling and softening of wood chips, resulting in lower necessary enzyme dosage and increased enzymatic digestibility [52]. Similarly, the disk milling energy of hot water-pretreated and superheated steam-pretreated oil palm mesocarp fiber were compared [56]. The hot water-pretreated sample and superheated steam-pretreated sample showed up to 22 and 73 % less milling energy, respectively, compared to the raw material sample. However, the hot water-pretreated sample had a high degree of viscosity, which led to higher milling energy consumption compared to the superheated steam-pretreated sample. Huo et al. [65] measured milling energy of magnesium hydroxide-impregnated and sodium hydroxide-impregnated eucalypt chips. The magnesium hydroxide-impregnated sample consumed 430 kWh milling energy per ton of biomass, while the sodium hydroxide-impregnated sample used 630 kWh/ton. Energy requirements for both pretreated eucalypt chips were lower compared to the non-chemical-pretreated sample (990 kWh/ton).

In addition to the type of hydrothermal/chemical pretreatment, many parameters affect milling energy, including biomass species, moisture content, feed rates, motor speed, and milling cycles. Zhu et al. [52] concluded that low solids loading in milling and large disk-plate gap decreased energy consumption without lowering glucose yields from enzymatic hydrolysis. When solid loading was decreased from 50 to 10 %, milling energy was reduced by 34 %. Milling energy was decreased by 80 to 90 % when the disk-plate gap was increased from 0.38 to 2.54 mm. Similarly, increasing throughput from 17.3 to 32.0 ton/day and plate gap from 0 to 1.78 mm decreased energy consumption from 468 to 128 kWh/ton [42]. Therefore, selecting the right type of hydrothermal/chemical pretreatment and optimizing milling conditions could significantly reduce milling energy and make the combined pretreatment feasible in the industrial application.

### Impact on physical structure of biomass

**Fig. 6:**
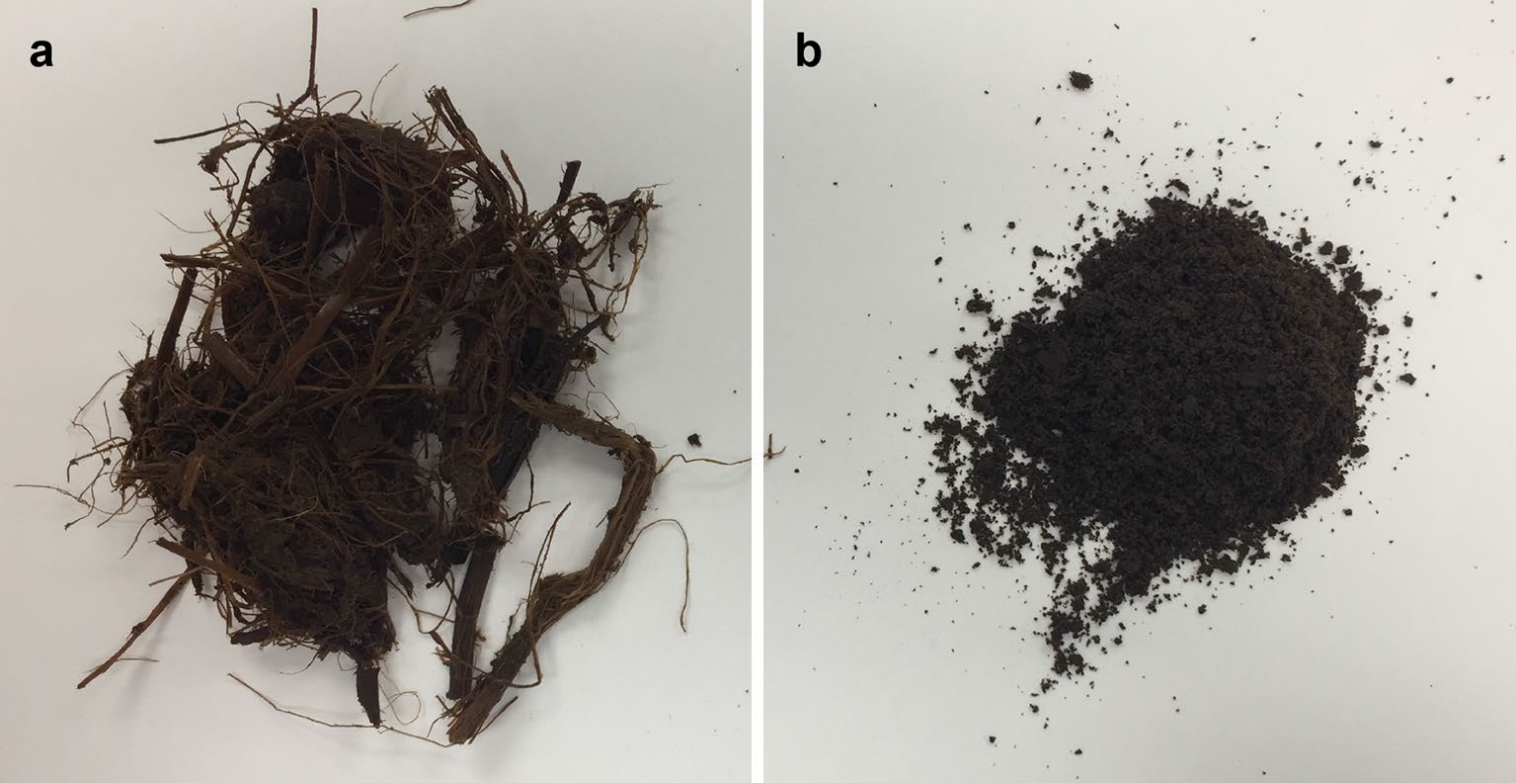
Size reduction of dilute acid-pretreated corn stover by disk milling. **a** Dilute acid-pretreated sample; **b** dilute acid-pretreated and disk-milled sample

Many factors affect enzymatic saccharification, including substrate size, specific surface area, accessibility to cellulase, crystallinity, lignin content, and structure. Combined hydrothermal/chemical pretreatment followed by mechanical refining reduces particle size, increases defiberization, decreases cellulose crystallinity, and increases accessible specific surface. Milling is effective in reducing particle size, which can be observed visually (Fig. 6). Chen et al. [49] measured acid-pretreated corn stover particle size after PFI milling and disk milling. From acid-pretreated sample average particle size of 270.7 μm, sizes of 83.7–95.5 μm were achieved after a PFI milling, while 139.3–163.8 μm were reached after disk milling. Ball milling can also reduce particle size. The average particle size of oil palm mesocarp fiber (407.5 μm) decreased to 233.8 μm after 240 min of ball milling [38]. Even 1 min of ball milling was effective to reduce the particle size of corn straw from 160.40 to 64.35 μm [66]. Interestingly, ball milling after ozone pretreatment decreased particle size to 88.28 μm, which was not as much as ball milling alone. However, the samples subjected to combined pretreatment achieved higher sugar yields compared to samples pretreated by ball milling alone.

Mechanical refining dramatically reduces particle size, but generally, particle size does not correlate with enzymatic sugar release [67, 68], which can be explained by the different types of size reduction. Leu and Zhu [68] categorized size reduction into two classes. Class I size reduction increases fiber external surface area by fiber separation, cutting, fragmentation, and external fibrillation by shear forces. Class I size reduction plays a minor role in increasing enzymatic digestibility. In Class II size reduction, cell walls are significantly deconstructed by breaking up microfibril cross-links and by compression-induced internal fibrillation. Class II size reduction can be achieved by disk milling, ball milling, extrusion, and PFI milling. Since Class II size reduction destroys the cell wall, it simultaneously reduces crystallinity. For example, sugarcane bagasse after alkaline pretreatment and disk milling showed lower crystallinity index (26 %) compared to samples after alkaline pretreatment alone (38 %) [63]. Ball milling can dramatically reduce crystallinity compared to disk milling. Hot water pretreatment followed by ball milling decreased the crystallinity of eucalyptus from 59.7 to 13.2 % [37]. Ozone-and-ball milling-treated corn straws also decreased crystallinity index from 48 to 4 % [66].

### Pilot/industrial-scale milling

Similar results as observed in lab-scale milling have been observed in pilot/industrial-scale milling. Sugar yields of deacetylated and dilute acid-pretreated corn stover were improved by 6 to 7 % after one or two passes of the Szego mill, a planetary ring-roller mill currently used at commercial scale [49]. After three passes through the Szego mill, the glucose and xylose yields reached around 95 % and 90 %, respectively, which were 10–11 % higher compared to non-refined samples. A small industrial-scale disk mill (Sprout model 401, 36 inch diameter) also improved sugar yields [42]. Non-refined deacetylated and dilute acid-pretreated corn stover showed 69 % glucose yield and 54 % xylose yield, while refining the sample increased glucose and xylose yields to 86 and 79 %, respectively.

The effects of combined pretreatment on sugar yield and energy consumption have also been observed on an industrial scale. Sodium carbonate-pretreated pulp was milled with two industrial mills consecutively [69]. First, milling was done by Beloit double-disk (42″ diameter) refiner with high-intensity plates. Milled fiber then passed through the secondary refiner, a 42 inch Sprout-Bauer twin flow refiner with mid-intensity plates. Non-refined samples yielded 26.30 % total sugar after 48 h enzymatic hydrolysis, and increases in the total sugar yields were observed after both the primary and the secondary refining. The primary refined samples achieved total sugar yields of 43.9 % after 48 h enzymatic hydrolysis, and energy consumption of the primary refiner was 67.0 kWh/ton. Only a small increase of total sugar yields (50.1 % after 48 h enzymatic hydrolysis) was seen for secondary refined samples. However, the secondary refiner consumed an additional 79.5 kWh/ton. To increase total sugar yields without the high energy demand of secondary refining, an alternative strategy is to perform longer enzymatic hydrolysis. Primary refined samples showed 62.5 % total sugar yields after 144 h enzymatic hydrolysis, which were higher than the secondary refined samples’ total sugar yields (50.1 %) after 48 h enzymatic hydrolysis [69].

Tao et al. [70] performed techno-economic analysis of deacetylated, dilute acid-pretreated, and mechanically refined samples based on the experimental data generated by Chen et al. [58], which was compared to a techno-economic analysis that was published by National Renewable Energy Laboratory (NREL) in 2011 [3]. The biggest difference between the 2011 and 2012 experimental designs is the dilute acid conditions. For the 2011 design, pretreatment were conducted for 5 min at 158 °C and 5.5 atm with 22 mg of sulfuric acid loading per gram of dry biomass. For the 2012 design, only 8 mg of sulfuric acid was added per gram of dry biomass, and pretreatment was performed for 20 min at 150 °C and 4 atm. In addition to dilute acid pretreatment, deacetylation and mechanical refining were evaluated in the 2012 techno-economic analysis. For the 2012 scenario, PFI milling after dilute acid pretreatment could reduce the minimum ethanol selling price (MESP) by $0.19 or $0.30 per gallon depending on corn stover varieties compared to dilute acid pretreatment alone. Moreover, samples that undergo deacetylation, dilute acid pretreatment and PFI milling could reduce MESP by $0.44 or $0.54 per gallon. Combining deacetylation, dilute acid pretreatment and mechanical refining could produce 64 million gallons of ethanol per year with $2.12 per gallon MESP, which represents 25 % higher ethanol yields and $0.03 lower MESP compared to the 2011 design. More techno-economic and life cycle analyses need to be conducted to prove the commercial feasibility of the combined pretreatment.

## Future perspectives

For the case of woody biomass, the series of papers on the SPORL process are comprehensive. The same cannot be claimed for thermal mechanical processing of herbaceous biomass. There are at least four major research gaps. The first is the lack of an energy balance and in particular a measure of electrical usage. The second gap is a mechanistic understanding of how thermomechanical systems lower enzyme loading while still achieving high product yields. Enzyme loading is determined by cell wall structure (or lack thereof) and by non-specific lignin binding. It would be of interest to determine if the lower temperature afforded by the subsequent mechanical refining lowers non-specific binding of cellulases. This result would have important consequences for enzyme recycling and operating costs. The third gap is to investigate if a two-stage process, with its reduced generation of inhibitors, will afford increased process efficiencies as measured by fermentation yields and net process water usage. The fourth is the scarcity of data on herbaceous biomass and absence of studies using perennial grasses. While combined chemical/thermomechanical refining is well established for the pulping industry, it represents a new but very promising area of research for biomass conversion because of its ability to increase enzymatic conversion at lower severities and the untested possibility to reduce net enzyme and water usage.

## Conclusions

Hydrothermal/chemical, physical, and biological pretreatments have their own unique mechanisms to destruct biomass cell wall structure. Combined pretreatment featuring hydrothermal/chemical pretreatment followed by mechanical refining showed synergistic effects to reduce particle size, crystallinity, and enzyme dosage and increase sugar yields compared to hydrothermal/chemical pretreatment alone or mechanical refining alone. In addition, energy consumption of mechanical refining can be decreased when preceded by hydrothermal/chemical pretreatment that modifies and swells the biomass structure. The combined pretreatment not only successfully increased sugar yields in lab scale, but also has demonstrated potential application in industrial scale.

## Abbreviations

AAS: aqueous ammonia soaking
AFEX: ammonia fiber explosion
ARP: ammonia recycled percolation
CBP: consolidated bioprocessing
CELF: co-solvent enhanced lignocellulosic fractionation
DOE: Department of Energy
FPU: filter paper unit
HSF: hybrid saccharification and fermentation
MESP: minimum ethanol selling price
NREL: National Renewable Energy Laboratory
PFI: Papirindustriens Forskningsinstitutt
SHF: separate hydrolysis and fermentation
SPORL: sulfite pretreatment to overcome recalcitrance of lignocellulose
SScF: simultaneous saccharification and co-fermentation
SSF: simultaneous saccharification and fermentation
