# Erratum to: Promise of combined hydrothermal/chemical and mechanical refining for pretreatment of woody and herbaceous biomass

**DOI:** 10.1186/s13068-016-0643-6

**Published:** 2016-12-06

**Authors:** Sun Min Kim, Bruce S. Dien, Vijay Singh

**Affiliations:** 1 Department of Agricultural and Biological Engineering, University of Illinois at Urbana-Champaign, Urbana, IL 61801 USA; 2 Bioenergy Research Unit, Agricultural Research Service, USDA, National Center for Agricultural Utilization Research, Peoria, IL 61604 USA

## Erratum to: Biotechnol Biofuels (2016) 9:97 DOI 10.1186/s13068-016-0505-2

Unfortunately, after publication of this article [1], it was noticed that the capturing of Table 4 during the production process introduced several items in the ‘Sugar Yields’ column listed in the wrong row. The corrected table can be seen in this erratum (Table 4).

**Table 4:** Comparison of hydrothermal/chemical pretreatment followed by mechanical refining and hydrothermal/chemical pretreatment alone or mechanical refining alone

Sample	Pretreatment^a^	Milling energy (kWh/ton)^b,c^	Sugar yield (%)^d^	Reference
Hardwood chips	Sodium carbonate	NA	42.11 (total sugar)	[53]
	Sodium carbonate + PFI milling	360–1800	46.90–53.12 (total sugar)	
	Sodium carbonate + disk milling (12 in. diameter)	698	69.51 (total sugar)	
	Sodium carbonate + disk milling (42 in. diameter)	67–147	62.48–66.51 (total sugar)	
Japanese cedar	Ozonolysis	NA	28–68 (glucose)	[54]
			29–44 (xylose)	
	Disk milling	4167–26,389	38–75 (glucose)	
			26–45 (xylose)	
	Ozonolysis + disk milling	8333–22,222	71–94 (glucose)	
			44–59 (xylose)	
Lodgepole pine trees	Disk milling	615.9	11.3 (glucose)	[52]
	Hot water (initial pH 5.0) + disk milling	537.0	33.1 (glucose)	
	Acid (initial pH 1.1) + disk milling	335.6	39.6 (glucose)	
	SPORL (initial pH 4.2) + disk milling	499.3	84.1 (glucose)	
	SPORL (initial pH 1.9) + disk milling	134.5	92.2 (glucose)	
Eucalyptus chips	Disk milling	990	72.94 (total sugar)	[65]
	Sodium hydroxide impregnation + disk milling	630	80.77 (total sugar)	
	Magnesium hydroxide impregnation + disk milling	430	91.53 (total sugar)	
Hinoki cypress	Disk milling	853	50 (glucose)	[74]
	Steam treatment + disk milling	744–1489	96.8 (glucose)	
Eucalyptus chips	Disk milling	408	45 (glucose)	
	Steam treatment + disk milling	192–458	98.4 (glucose)	
Eucalyptus chips	Hot water	NA	50 (glucose)	[55]
	Hot water + disk milling	167	101.7 (glucose)	
Eucalyptus chips	Hot water	NA	3.1–65.2 (total sugar)	[37]
	Hot water + ball milling	1436	45.6–66.7 (total sugar)	
Rice straw	Hot water	NA	97.5 (glucose)	[62]
	Hot water + mechanical refining	250–583	97.3–99.5 (glucose)	
Oil palm mesocarp fiber	Disk milling	5250	30.2 (glucose)	[56]
			30.6 (xylose)	
	Superheated steam + disk milling	1417–3028	26.0–47.8 (glucose)	
			24.1–42.1 (xylose)	
	Hot water + disk milling	4083–4972	46.3–91.1 (glucose)	
			10.1–54.3 (xylose)	
Corn stover	Alkali deacetylation + disk milling (36 in. diameter)	128–468	85.9–91.7 (glucose)	[42]
			81.1–86.2 (xylose)	
Sugarcane bagasse	Alkaline + disk milling	11,111	77 (glucose)	[63]
			67 (xylose)	
Sugarcane bagasse	Hot water	NA	72.1–78.7 (total sugar)	[75]
	Hot water + PFI refining		82.1–87.2 (total sugar)	
Wheat straw	Hot water	NA	28.1–72.4 (total sugar)	[59]
	Hot water + PFI refining		28.3–75.5 (total sugar)	
Oil palm mesocarp fiber	Ball milling	NR	7.3–10.3 (glucose)	[38]
			12.2–14.9 (xylose)	
	Alkaline		39.6–63.9 (glucose)	
			21.1–46.5 (xylose)	
	Alkaline + ball milling		97.3 (glucose)	
			63.2 (xylose)	
Corn stover	Acid impregnation + dilute acid	NA	69–73 (glucose)	[58]
			55–58 (xylose)	
	Alkali deacetylation + acid impregnation + dilute acid		80–83 (glucose)	
			76–80 (xylose)	
	Acid impregnation + dilute acid + PFI refining		85 (glucose)	
			75 (xylose)	
	Alkali deacetylation + acid impregnation + dilute acid + PFI refining		90 (glucose)	
			92 (xylose)	
Corn stover	Alkali deacetylation + acid impregnation + steam explosion + PFI refining	NR	79–83 (glucose)	[49]
			50–55 (xylose)	
	Alkali deacetylation + acid impregnation + steam explosion + extruder		82–83 (glucose)	
			56–58 (xylose)	
	Alkali deacetylation + acid impregnation + steam explosion + food processor/blending		71–75 (glucose)	
			49–51 (xylose)	
	Alkali deacetylation + acid impregnation + steam explosion + disk milling (12 in.)		75–78 (glucose)	
			52–54 (xylose)	
	Alkali deacetylation + acid impregnation + dilute acid (pilot-scale)		82 (glucose)	
			80 (xylose)	
	Alkali deacetylation + acid impregnation + dilute acid pretreatment (pilot-scale) + Szego milling		90–95 (glucose)	
			85–90 (xylose)	
Eucalyptus chips	Hot water	NA	73.19 (glucose)	[73]
			90.45 (xylose)	
	Hot water + disk milling		91.62 (glucose)	
			88.12 (xylose)	
Rice straw	Disk milling	NR	86 (glucose)	[60]
			40 (xylose)	
	Hot water + disk milling		110 (glucose)	
			84 (xylose)	

*NA* not applicable, *NR* not reported

^a^Hot compressed water, hydrothermal and autohydrolysis are named as hot water

^b^When energy consumption was presented as kJ/ton, it was converted into kWh/ton

^c^Energy consumption is only from mechanical refining

^d^If the exact sugar yields were not indicated in the reports, sugar yields were estimated or calculated as the ratio of the amount of monosaccharides produced during hydrolysis to the corresponding carbohydrate concentrations in the original samples
